# Prediction of 5‐year overall survival of diffuse large B‐cell lymphoma on the pola‐R‐CHP regimen based on 2‐year event‐free survival and progression‐free survival

**DOI:** 10.1002/cam4.6899

**Published:** 2024-01-05

**Authors:** Wan‐Ru Zhang, Xin Liu, Qiu‐Zi Zhong, Tao Wu, Yong Yang, Bo Chen, Hao Jing, Yuan Tang, Jing Jin, Yue‐Ping Liu, Yong‐Wen Song, Hui Fang, Ning‐Ning Lu, Ning Li, Yi‐Rui Zhai, Wen‐Wen Zhang, Shu‐Lian Wang, Fan Chen, Lin Yin, Shu‐Nan Qi, Ye‐Xiong Li

**Affiliations:** ^1^ National Cancer Center/National Clinical Research Center for Cancer/Cancer Hospital Chinese Academy of Medical Sciences (CAMS) and Peking Union Medical College (PUMC); Collaborative Innovation Center for Cancer Medicine Beijing China; ^2^ Beijing Hospital, National Geriatric Medical Center Beijing China; ^3^ Affiliated Hospital of Guizhou Medical University, Guizhou Cancer Hospital Guiyang Guizhou China; ^4^ Fujian Medical University Union Hospital Fuzhou Fujian China; ^5^ National Cancer Center/National Clinical Research Center for Cancer/Cancer Hospital & Shenzhen Hospital Chinese Academy of Medical Sciences (CAMS) and Peking Union Medical College (PUMC) Shenzhen China; ^6^ Affiliated Hospital of Qinghai University Qinghai China

**Keywords:** diffuse large B‐cell lymphoma, immunochemotherapy, overall survival prediction, pola‐R‐CHP

## Abstract

This study aimed to predict the 5‐year overall survival (OS) benefit of pola‐R‐CHP versus R‐CHOP in the POLARIX trial based on the 2‐year event‐free survival (EFS) and progression‐free survival (PFS) rates in diffuse large B‐cell lymphoma (DLBCL). We identified randomized controlled trials (RCT) published before 31 May 2023. The correlation between the logarithmic (log) hazard ratio (HR) for EFS (HR_EFS_) or PFS (HR_PFS_) and the HR for OS (HR_OS_) was estimated at the trial‐level. Correlation analysis was performed between 2‐year PFS or EFS and 5‐year OS rates at the treatment arm‐level. Linear regression models were used to calculate the 5‐year OS of pola‐R‐CHP and R‐CHOP. In the included 20 RCTs, a linear correlation between HR_EFS_ (*r* = 0.765) or HR_PFS_ (*r* = 0.534) and HR_OS_ was observed at the trial‐ level. Two‐year EFS (*r* = 0.918) or 2‐year PFS (*r* = 0.865) correlated linearly with 5‐year OS. Linear regression analysis between 2‐year EFS/PFS and 5‐year OS gave estimated 5‐year OS rates between pola‐R‐CHP and R‐CHOP of 6.4% and 6.3%, respectively. Two‐year EFS and PFS are feasible early endpoints in patients with DLBCL treated primarily with immunochemotherapy. The pola‐R‐CHP regimen is expected to improve 5‐year OS.

## 1 INTRODUCTION

Immunochemotherapy with rituximab plus cyclophosphamide, doxorubicin, vincristine, and prednisone (R‐CHOP) has been considered the standard first‐line treatment for diffuse large B‐cell lymphoma (DLBCL) in recent decades.^1^, ^2^, ^3^, ^4^ The prognosis of previously untreated patients with DLBCL has improved, with a 5‐year overall survival (OS) rate of 60%–95%. However, approximately 40% of patients develop disease progression or relapse.^5^ The unfavorable outcomes of patients with refractory/relapsed DLBCL demand more effective systemic regimens in the first‐line treatment setting.

In the era of rituximab‐based immunochemotherapy, multiple randomized controlled trials (RCT) have focused on the use of intensified/de‐escalated chemotherapy;^6^, ^7^, ^8^, ^9^, ^10^, ^11^, ^13^, ^14^, ^15^, ^16^, ^17^ the addition of maintenance or consolidation therapy^12^, ^18^, ^19^, ^20^, ^21^, ^22^, ^23^, ^24^ and novel targeted therapy;^25^, ^26^, ^27^ and the novel method of administering anti‐CD20 antibodies.^28^, ^29^ Except for one trial,^6^ none of these RCTs demonstrated a survival benefit with these new regimens compared with R‐CHOP. Recently, polatuzumab vedotin, an anti‐CD79b monoclonal antibody conjugated by a protease‐cleavable linker to monomethyl auristatin E (a potent microtubule inhibitor),^30^, ^31^ has been shown to have a high antitumor activity in B‐cell lymphoma.^32^, ^33^, ^34^ In 2022, the POLARIX trial demonstrated that pola‐R‐CHP (polatuzumab vedotin, rituximab, cyclophosphamide, doxorubicin, and prednisone) versus R‐CHOP significantly improved 2‐year event‐free survival (EFS) and progression‐free survival (PFS) rates in patients with intermediate‐ and high‐risk DLBCL.^32^ Despite the lack of OS benefit, probably because of the short follow‐up time of 2.4 years, the 2023 National Comprehensive Cancer Network (NCCN) guideline recommends pola‐R‐CHP as a first‐line regimen for high‐risk DLBCL.^35^ Before the publication of the mature results of POLARIX, there is an urgent need to estimate whether pola‐R‐CHP regimen would provide long‐term OS benefit.

Previous studies have confirmed that 2‐year EFS and PFS are surrogate endpoints for OS after immunochemotherapy for DLBCL at the trial‐and individual‐level.^36^, ^37^, ^38^, ^39^ Another early study also demonstrated that improvements in 3‐year EFS/PFS are highly correlated with improvements in 5‐year OS in aggressive non‐Hodgkin lymphoma.^40^ Furthermore, improved EFS and PFS are associated with prolonged OS at the treatmentarm level in DLBCL.^41^ Given that EFS and PFS was effective early efficacy endpoints in DLBCL, we hypothesized that 2‐year EFS and PFS might also serve as predictors of long‐term OS in the immunochemotherapy era, and allow for precise assessment of long‐term OS benefit. The aim of this study was to evaluate the correlations between EFS/PFS and OS and then predict the 5‐year OS benefit of pola‐R‐CHP versus R‐CHOP in the POLARIX trial (Tilly 2022).^32^

## 2 METHODS

### 2.1 Data sources and searches

All RCTs published before 31 May 2023 were included via a systematic literature search of PubMed, EMBASE, and the Cochrane Library, mainly using the search terms “DLBCL AND rituximab”. Simultaneously, we conducted a thorough literature search using all relevant synonyms to avoid missing any relevant publications. Formal publications and meeting abstracts were included. Two investigators (W.R.Z. and S.N.Q.) independently searched the relevant studies, and any discrepancies were settled in collaboration with a principal investigator (Y.X.L.).

### 2.2 Inclusion and exclusion criteria

After eliminating duplicate publications, we included articles and abstracts that met the following criteria: (1) Phase III RCTs reporting the long‐term survival of patients with DLBCL who received first‐line rituximab‐containing immunochemotherapy; (2) R‐CHOP or R‐CHOP‐like regimens as a controlled treatment arm in RCTs; (3) data available for 2‐year EFS/PFS and 5‐year OS rates extracted from studies directly or the Kaplan–Meier survival curve; and (4) patients with DLBCL consisted of >80% of the whole‐sample size. Studies were excluded if they met any of the following criteria: Phase I/II or retrospective studies; transformed or relapsed/refractory DLBCL; inadequate survival data; studies on serum positivity for HIV, hepatitis B/C virus, or Epstein–Barr virus; or a sample size of <90 patients per arm.

A total of 20 RCTs with 45 treatment arms and 12,141 patients met the eligibility criteria (Table 1), and were included in the final analysis.^6^, ^7^, ^8^, ^9^, ^10^, ^11^, ^12^, ^13^, ^14^, ^15^, ^16^, ^17^, ^18^, ^19^, ^20^, ^21^, ^22^, ^23^, ^24^, ^25^, ^29^ Each treatment arm consisted of 97–710 patients (median sample size: 249). Eight RCTs were excluded due to CHOP as standard treatment arm (*n* = 4) and lack of data on 5‐year OS in the short follow‐up time (*n* = 4) (Table S1).^1^, ^2^, ^3^, ^4^, ^26^, ^27^, ^28^, ^42^

**TABLE 1:** Summary of 20 Phase III randomized controlled trials on immunochemotherapy in trial‐ and treatment arm‐level analyses.

Trial	Inclusion Criteria	Median FU (years)	No.	Treatment	2‐year PFS (%)	HR	2‐year EFS (%)	HR	5‐year OS (%)	HR
R‐CHOP (like) versus R + intensified/de‐escalated chemotherapy (*n* = 11)										
LNH03‐2B^6^	Age 18–59; all stages; aaIPI = 1	3.7	196	**R‐ACVBP**	89.7	0.48^a^ ^,P^	83.6	0.56^a^ ^,P^	91.6	0.44^a^ ^,P^
			183	R‐CHOP	74.6		67.7		79.9	
ANZINTER3^7^	Age >65; stage II–IV; PS ≤3; “fit” in CGA	3.5	114	**R‐miniCEOP**	NA	NA	54.4	1.12^a^ ^,N^	63^a^	0.92^a^ ^,N^
			110	R‐CHOP	NA		56.7		62^a^	
LNH03‐6B^8^	Age 60–80; aaIPI ≥ 1	4.7	304	**R‐CHOP‐14**	62.8	0.99^a^ ^,N^	58.7	1.04 ^N^	65.8	0.96^a^ ^,N^
			298	R‐CHOP‐21	66.2		64.6		59.6	
NCT01793844^9^	Age ≥18; all stages; PS ≤ 3	3.8	349	**R‐CHOP‐14**	65.8	1.1^a^ ^,N^	NA	NA	74.4	0.98^a^ ^,N^
			353	R‐CHOP‐21	70.2		NA		73.6	
UK NCRI^10^	Age ≥18; stage IB–IV or bulky IA; PS ≤ 2	3.8	540	**R‐CHOP‐14**	74.7	0.94^a^ ^,N^	NA	NA	75.7	0.9^a^ ^,N^
			540	R‐CHOP‐21	74.7		NA		72.7	
DLCL04^11^	Age 18–65; DLBCL (88.7%) or FL 3b; Stage II–IV; aaIPI 2–3; PS ≤ 2	6	196 203	**R‐MegaCHOP** R‐CHOP	NA NA	NA	66^a^ 67^a^	1.04^a^ ^,N^ (FFS)	76^a^ 79^a^	1.14^a^ ^,N^
Alliance/CALGB 50303^13^	Age ≥18; stage II–IV DLBCL (92.9%) or stage I PMBCL; PS ≤ 2	5.2	241 250	**DA‐EPOCH‐R** R‐CHOP	78.9^a^ 75.5^a^	0.93^a^ ^,N^	NA NA	NA	77.5^a^ 78.5^a^	1.09^a^ ^,N^
FLYER^14^	Age 18–60; stage I–II; PS ≤ 1; tumor <7.5 cm	5.5	293	**4R‐CHOP+2R**	96.9	0.91^N^	89.7	1.06^N^	97^a^	0.85^a^ ^,N^
			295	6R‐CHOP	95.7		90.5		98^a^	
PETAL^15^	Age 18–80; PS ≤ 3; PET (−), DLBCL (82.7%)	4.5	100	**6R‐CHOP+2R**	77.7^a^	NA	72.6^a^	1.24^a^ ^,N^	76.03	0.96^a^ ^,N^
			97	6R‐CHOP	82.3^a^		78.9^a^		75.21	
NHL‐001^16^	Age 16–80; DLBCL (96.7%) or FL 3b; all stages; PS ≤ 2; stratified by age (16–60, 61–80)	3.8	134	**R‐CEOP90**	88.8^a^	0.44^a^ ^,P^	NA	NA	89.9	0.8^a^ ^,N^
			133	**R‐CEOP70**	77.4^a^	0.9^a^ ^,N^	NA	NA	87.7	1^a^ ^,N^
			133	R‐CHOP50	75.9^a^		NA		86.3	
			121	R‐CEOP70	67.1	1.09^a^ ^,N^	NA	NA	68.5	1.02^a^ ^,N^
			122	R‐CHOP50	69.9		NA		66.4	
HOVON‐84^17^	Age 18–65 and aaIPI 1–3; age 66–80 and aaIPI 0–3	7.7	288	**RR‐CHOP‐14**	66.4		64.2 (FFS)	0.79^a^ ^,N^	69^a^	0.79^a^ ^,N^
			286	R‐CHOP‐14	71	0.83^a^ ^,N^	70.7 (FFS)		77^a^	
R‐CHOP (like) chemotherapy followed by maintenance/consolidation therapy (*n* = 8)										
AGMT‐NHL13^18^	Age >18; all stages; PS ≤ 2; CR/CRu	3.8	338 345	**R‐CHOP‐like+R** R‐CHOP‐like	88.5 84.1	0.62^a^ ^,P^	83.7 81.5	0.79^a^ ^,N^	90.7 88.3	0.81^a^ ^,N^
PRELUDE^19^	Age ≥18; bulky II/III–IV; IPI ≥ 3; PS ≤ 2; CR/CRu	4	493 249	**R‐CHOP+enzastaurin** R‐CHOP	78.1^a^ 73.4^a^	0.9^a^ ^,N^ (DFS)	NA NA	NA	76.4 75.6	1.04^a^ ^,N^
REMARC^20^	Age 60–80; Stage II–IV; aaIPI ≥ 1; PS ≤ 2; CR or PR	3.3^b^	323	**R‐CHOP+lenalidomide**	80^a^	0.71^a^ ^,P^	NA	NA	76.2	1.22^a^ ^,N^
			327	R‐CHOP	75^a^		NA		77.7	
PILLAR‐2^21^	Age >18; stage II (>10 cm) or III–IV; IPI ≥ 3; CR	4.2	372 370	**R‐chemo+everolimus** R‐chemo	77.8^a^ 77^a^	0.92^a^ ^,N^ (DFS)	NA NA	NA	79.2 72.5	0.75^a^ ^,N^
DSHNHL2002‐1^12^, ^22^	Age 18–60; aaIPI 2–3; CD20‐positive B‐cell lymphoma (DLBCL 80.5%); PS ≤ 3	9.3	132 130	**R‐MegaCHOP** R‐CHOP	71.8 77.5	1.1^a^ ^,N^	63.1 71.7	1.3^a^ ^,N^	73.3 82.3	1.3^a^ ^,N^
DLCL04^11^	Age 18–65; DLBCL (88.7%) or FL 3b; aaIPI 2–3; PS ≤ 2	6	199 200	R‐HDC+ASCT **No ASCT**	72^a^ 65^a^	0.72^a^ ^,N^	71^a^ 62^a^	0.65^a^ ^,P^ (FFS)	78^a^ 77^a^	0.98^a^ ^,N^
NCT00355199^23^	Age 18–65; stage III–IV or bulky II; high risk	5	113	**R‐CHOP+ASCT**	74.4	0.84^N^	65.2	0.99^a^ ^,N^	76.8	0.95^a^ ^,N^
			122	R‐CHOP	66.5		62.4		71.7	
LYSA/GOELAMS^24^	Age 18–75; stage I–II; tumor <7 cm	5.3	165	**R‐CHOP+RT**	NA	NA	97.3	0.61^a^ ^,N^	96^a^	0.62^a^ ^,N^
			169	R‐CHOP	NA		93.3		92^a^	
R‐CHOP + novel targeted drug (*n* = 1)										
REMoDL‐B^25^	Age ≥18; stage I (>10 cm) or II–IV; PS ≤ 2; with GEP	5.3	394 407	**RB‐CHOP** R‐CHOP	75.1^a^ 71.0^a^	0.81^a^ ^,N^	NA NA	NA	79.4^a^ 75.9^a^	0.86^a^ ^,N^
Anti‐CD20 monoclonal antibody study (*n* = 1)										
GOYA^29^	Age ≥18; >1.5 cm; PS ≤ 2; IPI 0 (>7.5 cm) or 1 (age <60) or ≥2	4	704 710	**G‐CHOP** R‐CHOP	73 70.8	0.94^a^ ^,N^	NA NA	0.95^a^ ^,N^	77^a^ 77.7^a^	1.02^a^ ^,N^

*Notes*: The standard arm is labeled in bold. “P” and “N” in the top right of the HR indicate positive and negative results, respectively. Trials: CALGB/Alliance 50303, Cancer and Leukemia Group B/Alliance 50303; DSHNHL2002‐1, German High‐Grade Non‐Hodgkin Lymphoma Study Group 2002‐1; LYSA/GOELAMS, Lymphoma Study Association/Groupe Ouest‐Est d'études des Leucémies Aigües et autres Maladies du Sang; PETAL, PET‐Guided Therapy of Aggressive NHLs. Chemotherapy regimens: CEOP, cyclophosphamide, epirubicin, vinblastine, and prednisone; CHOEP, cyclophosphamide, doxorubicin, vincristine, etoposide, and prednisone; CHOP, cyclophosphamide, doxorubicin, vincristine, and prednisone; DA‐EPOCH‐R, dose‐adjusted etoposide, prednisone, vincristine sulfate, doxorubicin hydrochloride, cyclophosphamide, and rituximab; G‐CHOP, obinutuzumab, cyclophosphamide, doxorubicin, vincristine, and prednisone; R, rituximab; R‐ACVBP, rituximab, doxorubicin, cyclophosphamide, vindesine, bleomycin, and prednisone; R‐chemo, rituximab‐based chemotherapy; R‐CEOP70, rituximab, cyclophosphamide, epirubicin (70 mg/m^2^), vincristine, and prednisone; R‐CEOP90, rituximab, cyclophosphamide, epirubicin (90 mg/m^2^), vincristine, and prednisone; R‐CHOEP, rituximab, cyclophosphamide, doxorubicin, vincristine, etoposide, and prednisone; R‐CHOP, rituximab, cyclophosphamide, doxorubicin, vincristine, and prednisone; R‐CHOP‐14, R‐CHOP every 14 days; R‐CHOP‐21, R‐CHOP every 21 days; R‐CHOP50, rituximab, cyclophosphamide, doxorubicin (50 mg/m^2^), vincristine, and prednisone; R‐HDC, rituximab and high‐dose chemotherapy; R‐MegaCHOEP, R‐CHOEP with escalated doses of cyclophosphamide, etoposide, and doxorubicin; R‐miniCEOP, rituximab, cyclophosphamide, epirubicin, vinblastine, and prednisone; RB‐CHOP, R‐CHOP with bortezomib.

Abbreviations: aaIPI, age‐adjusted International Prognostic Index; ASCT, autologous stem cell transplantation; CGA, comprehensive geriatric assessment; CR, complete response; CRu, unconfirmed CR; DFS, disease‐free survival; DLBCL, diffuse large B‐cell lymphoma; EFS, event‐free survival; FFS, failure‐free survival; FL, follicular lymphoma; GEP, gene expression profiling; HR, hazard ratio; IPI, International Prognostic Index; NA, not available; NHL, non‐Hodgkin lymphoma; OS, overall survival; PET, positron emission tomography; PFS, progression‐free survival; PMBCL, primary mediastinal large B‐cell lymphoma; PR, partial response; PS, performance status; RT, radiotherapy; FU, median follow‐up; y, year; No., number of patients.

^a^ Represents data directly reported in the full text.

^b^ PFS, median follow‐up of 3.3 years; OS, median follow‐up of 4.3 years. [Correction added on January 10, 2024 after first online publication. The table 1 orientation has been changed to landscape in this version.]

### 2.3 Quality assessment

The quality of the included RCTs were assessed using the Cochrane risk of bias tool with six domains: random sequence generation, allocation concealment, blinding of participants and personnel, blinding of outcome assessment, incomplete outcome data, and selective reporting (Figure S1). Ultimately, 20 RCTs were included in the analyses (Table 1).^6^, ^7^, ^8^, ^9^, ^10^, ^11^, ^12^, ^13^, ^14^, ^15^, ^16^, ^17^, ^18^, ^19^, ^20^, ^21^, ^22^, ^23^, ^24^, ^25^, ^29^

### 2.4 Endpoint definition

In these RCTs,^6^, ^7^, ^8^, ^9^, ^10^, ^11^, ^12^, ^13^, ^14^, ^15^, ^16^, ^17^, ^18^, ^19^, ^20^, ^21^, ^22^, ^23^, ^24^, ^25^, ^29^ OS was defined as the time from randomization to death from any cause. Generally, EFS was defined as the period from randomization to any treatment failure, including disease progression, death, and treatment discontinuity for any reason (e.g., adverse effects or withdrawal); PFS was measured from the time of randomization to disease progression, relapse, or death from any cause.

### 2.5 Data extraction

The detailed data of all included studies were extracted using a standardized form, including first author's name, year of publication, inclusion criteria, median follow‐up time, number of patients in each treatment arm, 2‐year EFS/PFS rate, 5‐year OS rate, and hazard ratios (HRs). Survival rates and HRs were extracted from the included studies directly (labeled “*” in Table 1) or the Kaplan–Meier survival curve using Engauge Digitizer software.^43^ For a repeatedly reported RCT, the latest results with the longest follow‐up time were selected.

### 2.6 Statistical analysis

The correlation between the logarithmic (log) HR for EFS (HR_EFS_) or PFS (HR_PFS_) and the log HR for OS (HR_OS_) was estimated using the Pearson correlation coefficient, *r*, in weighted linear regression at the trial‐level. The correlation between the 2‐year EFS or PFS rate and the 5‐year OS rate was also evaluated using *r* in weighted linear regression (with the weight equal to sample size) at the treatment arm‐level. A value of *r* close to 1 indicated a strong correlation. The 5‐year OS rates of the two treatments in the POLARIX trial were calculated using these two linear regression models. To assess the consistency and stability of the developed predictive models in different treatment settings, sensitivity analyses were performed by leaving out each subgroup of trials in turn. All statistical analyses were performed using SPSS (version 27.0, IBM Corp., Armonk, NY, USA). Data visualization was performed using the ggplot2 package in *R* software (version 4.2.3, R Foundation for Statistical Computing).

## 3 RESULTS

### 3.1 Baseline characteristics of the included studies

The median follow‐up time ranged from 3.3 to 9.3 years (Table 1). Eleven RCTs (55%) reported one pair of HR_EFS_ and HR_OS_; one RCT reported two pairs of HR_EFS_ and HR_OS_. Sixteen RCTs (80%) reported one pair of HR_PFS_ and HR_OS_; one RCT reported three pairs of HR_PFS_ and HR_OS_. Moreover, 2‐year EFS rates were extracted from 11 RCTs with 24 treatment arms, whereas 2‐year PFS rates were extracted from 18 (95%) RCTs with 39 treatment arms.

### 3.2 Correlation between HR_EFS_ or HR_PFS_ and HR_OS_ at the trial‐ level

**FIGURE 1:**
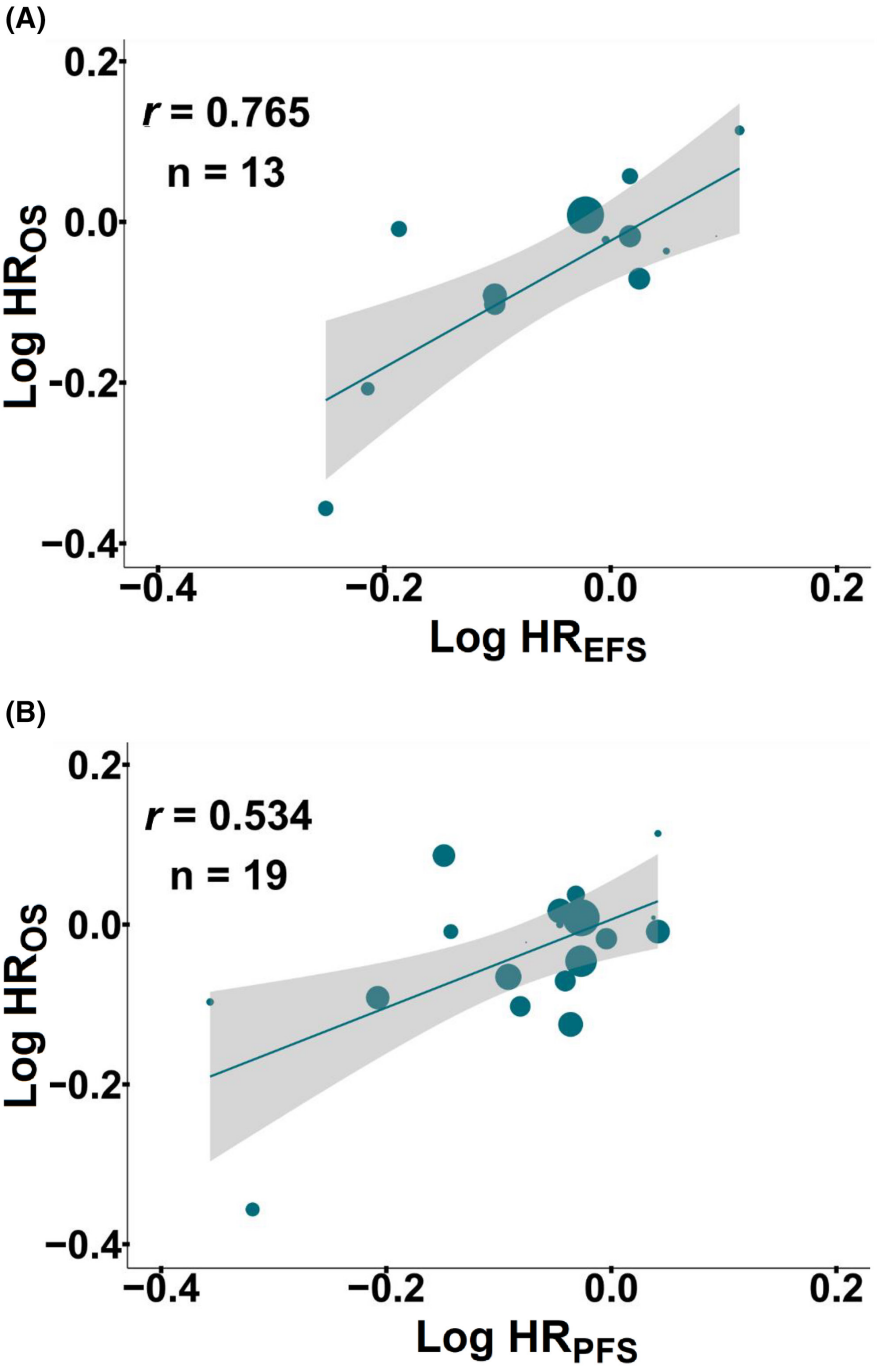
**Trial‐level correlation between treatment effects on EFS or PFS and OS in RCTs.** Trial‐level correlations between HR for EFS and HR for OS (A), and HR for PFS and HR for OS (B). The circle size is proportional to the number of patients in each comparison. The solid blue line indicates the fitted weighted linear regression line; the light gray zone represents its 95% CI. CI, confidence interval; EFS, event‐free survival; HR, hazard ratio; OS, overall survival; PFS, progression‐free survival; *r*, correlation coefficient; RCTs, randomized controlled trials.

We first determined the treatment effects of EFS and PFS on OS in RCTs in the setting of immunochemotherapy. At the trial level, there was a strong correlation between 13 pairs of log HR_EFS_ and log HR_OS_ (*r* = 0.765; 95% CI: 0.754–0.775; Figure 1A). The analysis of 19 pairs of log HR_PFS_ and log HR_OS_ demonstrated a moderate correlation (*r* = 0.534; 95% CI: 0.521–0.547; Figure 1B). This finding indicated that the treatment benefit in terms of EFS or PFS correlated with OS benefit at the trial‐level. Sensitivity analyses showed good consistency in most subgroups (Table 2).

**TABLE 2:** Summary of the sensitivity analyses.

Excluded subgroup	*r*	95% CI
Correlation between log HR (EFS) and log HR (OS)		
R + intensified/de‐escalated chemotherapy excluded	0.796	0.783–0.808
Maintenance/consolidation therapy excluded	0.840	0.831–0.849
Anti‐CD20 monoclonal antibody excluded	0.765	0.753–0.777
Correlation between log HR (PFS) and log HR (OS)		
R + intensified/de‐escalated chemotherapy excluded	0.208	0.183–0.232
Maintenance/consolidation therapy excluded	0.777	0.768–0.786
Novel targeted drug excluded	0.532	0.518–0.545
Anti‐CD20 monoclonal antibody excluded	0.516	0.501–0.530
Correlation between EFS (%) and OS (%)		
R + intensified/de‐escalated chemotherapy excluded	0.964	0.961–0.967
Maintenance/consolidation therapy excluded	0.929	0.924–0.934
Correlation between PFS (%) and OS (%)		
R + intensified/de‐escalated chemotherapy excluded	0.719	0.706–0.731
Maintenance/consolidation therapy excluded	0.893	0.889–0.898
Novel targeted drug excluded	0.868	0.863–0.872
Anti‐CD20 monoclonal antibody excluded	0.878	0.873–0.882

Abbreviations: CI, confidence intervals; EFS, event‐free survival; HR, hazard ratio; Log, logarithmic; OS, overall survival; PFS, progression‐free survival; *r*, correlation coefficient.

### 3.3 Linear correlation between 2‐year PFS or EFS rates and 5‐year OS rates at the treatment arm level

**FIGURE 2:**
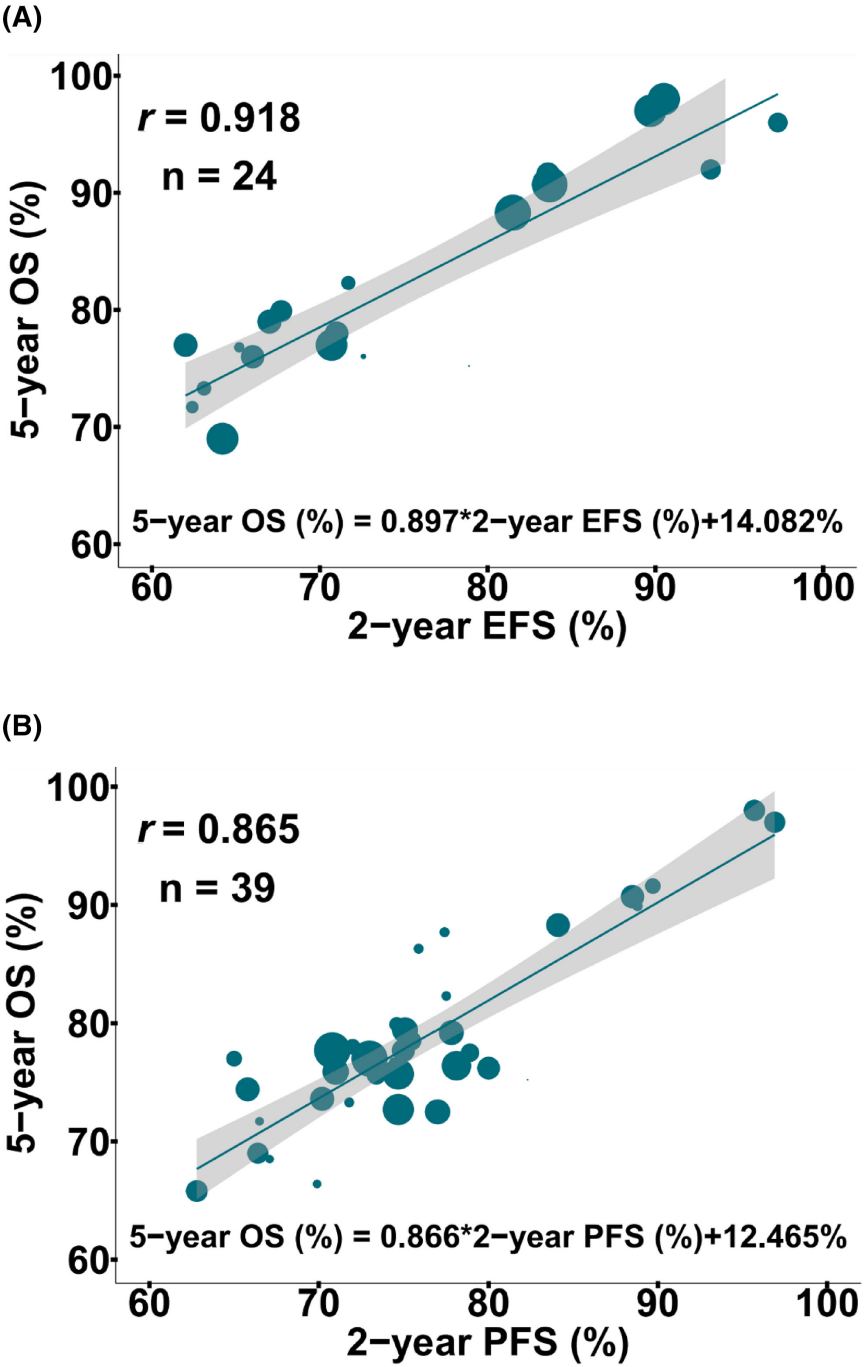
**Rituximab immunochemotherapy arm‐level correlation between 2‐year EFS or PFS and 5‐year OS in RCTs.** The rituximab immunochemotherapy arm‐level associations between 2‐year EFS and 5‐year OS (A), and 2‐year PFS and 5‐year OS (B). The circle size is proportional to the number of patients in each treatment arm. The solid blue line indicates the fitted weighted linear regression line; the light gray zone represents its 95% CI. CI, confidence interval. EFS, event‐free survival; OS, overall survival; PFS, progression‐free survival; *r*, correlation coefficient; RCTs, randomized controlled trials.

We further analyzed the correlation between 2‐year PFS or EFS rates and 5‐year OS rates, and established two linear regression models. At the treatment arm level, 2‐year EFS (*r* = 0.918; 95% CI: 0.913–0.922; Figure 2A) or 2‐year PFS (*r* = 0.865; 95% CI: 0.861–0.870; Figure 2B) correlated linearly with 5‐year OS. The linear regression models of EFS/PFS and OS were as follows: 5‐year OS (%) = 0.897 × 2‐year EFS + 14.082% (Figure 2A), and 5‐year OS (%) = 0.866 × 2‐year PFS + 12.465% (Figure 2B). As estimated from the EFS/PFS predictive models, an absolute gain of 10% 2‐year EFS and PFS provides 8.97% and 8.66% improvements in 5‐year OS, respectively. This finding indicated that improvement in 2‐year EFS or PFS is associated with increased 5‐year OS probabilities.

Sensitivity analyses showed good consistency in all subgroups. There was always a strong correlation between 2‐year PFS or EFS and 5‐year OS at the treatment arm‐level, regardless of included subgroups (Table 2).

### 3.4 Predicted 5‐year OS rates for the POLARIX trial using linear regression models

Using the EFS linear regression model, the predicted 5‐year OS rates for treatment with pola‐R‐CHP versus treatment with R‐CHOP for the intention‐to‐treat population were 77.4% versus 71%, respectively (Table 3). Similarly, based on the PFS linear regression model, patients treated with pola‐R‐CHP (74.7%) showed better 5‐year OS than those treated with R‐CHOP (68.4%) (Table 3). The absolute differences in the predicted 5‐year OS rates between the two treatment groups were 6.4% and 6.3% using the EFS and PFS linear regression models, respectively.

**TABLE 3:** Predicted 5‐year OS rates by 2‐year EFS and PFS rates.

	OS predicted by early EFS		OS predicted by early PFS	
Treatment‐arm	Reported 2‐year EFS (%)	Predicted 5‐year OS by EFS (%)	Reported 2‐year PFS (%)	Predicted 5‐year OS by PFS (%)
Pola‐R‐CHP	75.6	77.4	76.7	74.7
R‐CHOP	69.4	71	70.2	68.4
Absolute difference (%)	6.2	6.4	6.5	6.3

Abbreviations: EFS, event‐free survival; OS, overall survival; PFS, progression‐free survival; pola‐R‐CHP, polatuzumab vedotin, rituximab, cyclophosphamide, doxorubicin, and prednisone; R‐CHOP, rituximab, cyclophosphamide, doxorubicin, vincristine, and prednisone.

## 4 DISCUSSION

Our study demonstrated the feasibility of EFS and PFS as early endpoints in patients with DLBCL primarily treated with immunochemotherapy at the trial‐level. Moreover, the linear association of 2‐year EFS/PFS with 5‐year OS was confirmed at the treatment arm‐level, providing two predictive models. The predicted 5‐year OS differences were consistently ~6% with pola‐R‐CHP versus R‐CHOP in the POLARIX trial. The results of our study provide a practically and valuable suggestion for the application of pola‐R‐CHP to treat newly diagnosed DLBCL.

After the introduction of rituximab, which showed improved results for DLBCL, a ceiling effect of first‐line rituximab‐based immunochemotherapy has been observed over the last decade,^6^, ^7^, ^8^, ^9^, ^10^, ^11^, ^12^, ^13^, ^14^, ^15^, ^16^, ^17^, ^18^, ^19^, ^20^, ^21^, ^22^, ^23^, ^24^, ^25^, ^26^, ^27^, ^28^, ^29^ until the emergence of polatuzumab vedotin. Currently, two antibody drug conjugates (polatuzumab vedotin and loncastuximab tesirine) have been approved for previously untreated or relapsed/refractory DLBCL.^44^, ^45^, ^46^ The pilot study from the POLARIX trial demonstrated 2‐year EFS and PFS benefits of pola‐R‐CHP compared with R‐CHOP in patients with intermediate‐ and high‐risk DLBCL.^32^ The potent therapeutic efficacy of pola‐R‐CHP might be attributed to the characteristics of the highly specific targeting ability and potent killing effect of polatuzumab vedotin. However, with a median follow‐up time of 2.4 years, the OS benefit was not confirmed in the POLARIX trial.^32^ Although 2‐year EFS and PFS rates are widely used as surrogate endpoints in RCTs for DLBCL, it remains unclear whether early efficacy endpoints could predict long‐term OS. In this study, we further demonstrated the linear correlations of 2‐year EFS/PFS with 5‐year OS at both the trial‐ and treatment arm‐ level, indicating the association between improved 2‐year EFS or PFS and increased long‐term OS probabilities. Based on the EFS/PFS predictive models, the pola‐R‐CHP regimen is expected to result in a 5‐year OS improvement of approximately 6% compared with that of R‐CHOP. This finding provides additional data supporting the recommendation of pola‐R‐CHP in the current NCCN guidelines.^35^

The strengths of this study include strict inclusion criteria with uniform control treatment arms, a relatively large sample size from the RCTs, and an innovative approach to predicting OS rates. First, the data were obtained from high‐quality RCTs with strict screening processes according to our inclusion criteria. Our study enrolled a relatively large cohort (>12,000) of patients with newly diagnosed DLBCL treated with rituximab‐containing immunochemotherapy. The enrolled RCTs on immunochemotherapy with R‐CHOP or R‐CHOP‐like regimens as a controlled treatment arm for analysis made the linear regression models more applicable in the modern treatment era. Second, applying linear regression models to predict OS rates is unique in a specific RCT. If the predictive 5‐year OS rate and absolute difference between the two treatment arms are proven in the subsequent individual data analysis, the established 2‐year PFS and EFS linear regression models for DLBCL could be widely used in clinical practice. Any attempt at applying predictive model to predict OS rate of a specific RCT has the potential to assist in the design of future clinical trials and the interpretation of interim trial data.

Our study has several limitations. First, the definition of PFS and EFS events and the requirement for follow‐up were inconsistent across the included RCTs. In addition, the exact date of disease progression was dependent on the interval between two consecutive follow‐up visits, which could result imprecise dates in clinical practice. Such an inherent heterogeneity cannot be removed nor quantified in the linear regression analysis. Second, the establishment of the linear regression models in this study did not take into account information regarding post‐progression treatment because of the lack of data on salvage treatment in these RCTs. Over the last 6 years, the salvage therapy options for DLBCL has dramatically changed with the availability of CAR‐T cells and bispecific monoclonal antibodies.^47^, ^48^, ^49^, ^50^, ^51^ The OS improvements with novel salvage therapy strategy may alter the effect of first line therapy in DLBCL, leading to the decreased predictive ability of predicted models for OS. Upon the enhanced efficacy of salvage treatment or the availability of additional RCTs reporting salvage treatment data, the linear regression models will be further modified and optimized. However, it would be still very important to cure DLBCL patients with first‐line therapy as these salvage therapies are more complex and expensive, and have potentially serious side effects. Third, because this is a literature‐based predictive study without individual patient data, it is impossible to make a final conclusion until the long‐term OS in the POLARIX trial is published.

In conclusion, using the established linear regression models of 2‐year EFS/PFS and 5‐year OS in the modern treatment era, it is suggested that the pola‐R‐CHP regimen for intermediate‐ and high‐risk DLBCL would improve the 5‐year OS by approximately 6% compared with using R‐CHOP.

## AUTHOR CONTRIBUTIONS

**Wan‐Ru Zhang:** Data curation (equal); formal analysis (equal); methodology (lead); writing – original draft (lead). **Xin Liu:** Data curation (equal); formal analysis (equal); writing – original draft (supporting). **Qiuzi Zhong:** Data curation (equal); formal analysis (equal); writing – original draft (supporting). **Tao Wu:** Supervision (supporting); writing – review and editing (supporting). **Yong Yang:** Supervision (supporting); writing – review and editing (supporting). **Bo Chen:** Supervision (supporting); writing – review and editing (supporting). **Hao Jing:** Supervision (supporting); writing – review and editing (supporting). **Yuan Tang:** Supervision (supporting); writing – review and editing (supporting). **Jing Jin:** Supervision (supporting); writing – review and editing (supporting). **Yue‐Ping Liu:** Supervision (supporting); writing – review and editing (supporting). **Yong‐wen Song:** Supervision (supporting); writing – review and editing (supporting). **Hui Fang:** Supervision (supporting); writing – review and editing (supporting). **Ning‐Ning Lu:** Supervision (supporting); writing – review and editing (supporting). **Ning Li:** Supervision (supporting); writing – review and editing (supporting). **Yi‐Rui Zhai:** Supervision (supporting); writing – review and editing (supporting). **Wen‐Wen Zhang:** Supervision (supporting); writing – review and editing (supporting). **Shu‐Lian Wang:** Supervision (supporting); writing – review and editing (supporting). **Fan Chen:** Supervision (supporting); writing – review and editing (supporting). **Lin Yin:** Supervision (supporting); writing – review and editing (supporting). **Shu‐nan Qi:** Conceptualization (equal); funding acquisition (supporting); supervision (equal); writing – review and editing (supporting). **Ye‐xiong Li:** Conceptualization (equal); funding acquisition (lead); supervision (equal); writing – review and editing (lead).

## CONFLICT OF INTEREST STATEMENT

The authors declare no competing financial interests.

## Supporting information


Figure S1.

Table S1.

## Data Availability

The datasets used and/or analyzed during the current study are available from the corresponding authors on reasonable request.

## References

[1] Coiffier B , Thieblemont C , Van Den Neste E , et al. Long‐term outcome of patients in the LNH‐98.5 trial, the first randomized study comparing rituximab‐CHOP to standard CHOP chemotherapy in DLBCL patients: a study by the Groupe d'Etudes des Lymphomes de l'Adulte. Blood. 2010;116(12):2040‐2045. 20548096 10.1182/blood-2010-03-276246 PMC2951853

[2] Pfreundschuh M , Kuhnt E , Trümper L , et al. CHOP‐like chemotherapy with or without rituximab in young patients with good‐prognosis diffuse large‐B‐cell lymphoma: 6‐year results of an open‐label randomised study of the MabThera International Trial (MInT) Group. Lancet Oncol. 2011;12(11):1013‐1022. 21940214 10.1016/S1470-2045(11)70235-2

[3] Habermann TM , Weller EA , Morrison VA , et al. Rituximab‐CHOP versus CHOP alone or with maintenance rituximab in older patients with diffuse large B‐cell lymphoma. J Clin Oncol. 2006;24(19):3121‐3127. 16754935 10.1200/JCO.2005.05.1003

[4] Pfreundschuh M , Schubert J , Ziepert M , et al. Six versus eight cycles of bi‐weekly CHOP‐14 with or without rituximab in elderly patients with aggressive CD20+ B‐cell lymphomas: a randomised controlled trial (RICOVER‐60). Lancet Oncol. 2008;9(2):105‐116. 18226581 10.1016/S1470-2045(08)70002-0

[5] Sehn LH , Salles G . Diffuse large B‐cell lymphoma. N Engl J Med. 2021;384(9):842‐858. 33657296 10.1056/NEJMra2027612 PMC8377611

[6] Récher C , Coiffier B , Haioun C , et al. Intensified chemotherapy with ACVBP plus rituximab versus standard CHOP plus rituximab for the treatment of diffuse large B‐cell lymphoma (LNH03‐2B): an open‐label randomised phase 3 trial. Lancet. 2011;378(9806):1858‐1867. 22118442 10.1016/S0140-6736(11)61040-4

[7] Merli F , Luminari S , Rossi G , et al. Cyclophosphamide, doxorubicin, vincristine, prednisone and rituximab versus epirubicin, cyclophosphamide, vinblastine, prednisone and rituximab for the initial treatment of elderly "fit" patients with diffuse large B‐cell lymphoma: results from the ANZINTER3 trial of the Intergruppo Italiano Linfomi. Leuk Lymphoma. 2012;53(4):581‐588. 21895543 10.3109/10428194.2011.621565

[8] Delarue R , Tilly H , Mounier N , et al. Dose‐dense rituximab‐CHOP compared with standard rituximab‐CHOP in elderly patients with diffuse large B‐cell lymphoma (the LNH03‐6B study): a randomised phase 3 trial. Lancet Oncol. 2013;14(6):525‐533. 23578722 10.1016/S1470-2045(13)70122-0

[9] Li X , Huang H , Xu B , et al. Dose‐dense rituximab‐CHOP versus standard rituximab‐CHOP in newly diagnosed Chinese patients with diffuse large B‐cell lymphoma: a randomized, multicenter, open‐label phase 3 trial. Cancer Res Treat. 2019;51(3):919‐932. 30282447 10.4143/crt.2018.230 PMC6639223

[10] Cunningham D , Hawkes EA , Jack A , et al. Rituximab plus cyclophosphamide, doxorubicin, vincristine, and prednisolone in patients with newly diagnosed diffuse large B‐cell non‐Hodgkin lymphoma: a phase 3 comparison of dose intensification with 14‐day versus 21‐day cycles. Lancet. 2013;381(9880):1817‐1826. 23615461 10.1016/S0140-6736(13)60313-X

[11] Chiappella A , Martelli M , Angelucci E , et al. Rituximab‐dose‐dense chemotherapy with or without high‐dose chemotherapy plus autologous stem‐cell transplantation in high‐risk diffuse large B‐cell lymphoma (DLCL04): final results of a multicentre, open‐label, randomised, controlled, phase 3 study. Lancet Oncol. 2017;18(8):1076‐1088. 28668386 10.1016/S1470-2045(17)30444-8

[12] Frontzek F , Ziepert M , Nickelsen M , et al. Rituximab plus high‐dose chemotherapy (MegaCHOEP) or conventional chemotherapy (CHOEP‐14) in young, high‐risk patients with aggressive B‐cell lymphoma: 10‐year follow‐up of a randomised, open‐label, phase 3 trial. Lancet Haematol. 2021;8(4):e267‐e277. 33667420 10.1016/S2352-3026(21)00022-3

[13] Bartlett NL , Wilson WH , Jung SH , et al. Dose‐adjusted EPOCH‐R compared with R‐CHOP as frontline therapy for diffuse large B‐cell lymphoma: clinical outcomes of the phase III intergroup trial alliance/CALGB 50303. J Clin Oncol. 2019;37(21):1790‐1799. 30939090 10.1200/JCO.18.01994 PMC6774813

[14] Poeschel V , Held G , Ziepert M , et al. Four versus six cycles of CHOP chemotherapy in combination with six applications of rituximab in patients with aggressive B‐cell lymphoma with favourable prognosis (FLYER): a randomised, phase 3, non‐inferiority trial. Lancet. 2019;394(10216):2271‐2281. 31868632 10.1016/S0140-6736(19)33008-9

[15] Dührsen U , Müller S , Hertenstein B , et al. Positron emission tomography‐guided therapy of aggressive non‐Hodgkin lymphomas (PETAL): a multicenter, randomized phase III trial. J Clin Oncol. 2018;36(20):2024‐2034. 29750632 10.1200/JCO.2017.76.8093

[16] Xu PP , Fu D , Li JY , et al. Anthracycline dose optimisation in patients with diffuse large B‐cell lymphoma: a multicentre, phase 3, randomised, controlled trial. Lancet Haematol. 2019;6(6):e328‐e337. 31126528 10.1016/S2352-3026(19)30051-1

[17] Lugtenburg PJ , de Nully Brown P , van der Holt B , et al. Rituximab‐CHOP with early rituximab intensification for diffuse large B‐cell lymphoma: a randomized phase III trial of the HOVON and the Nordic Lymphoma Group (HOVON‐84). J Clin Oncol. 2020;38(29):3377‐3387. 32730183 10.1200/JCO.19.03418

[18] Jaeger U , Trneny M , Melzer H , et al. Rituximab maintenance for patients with aggressive B‐cell lymphoma in first remission: results of the randomized NHL13 trial. Haematologica. 2015;100(7):955‐963. 25911553 10.3324/haematol.2015.125344 PMC4486230

[19] Crump M , Leppä S , Fayad L , et al. Randomized, double‐blind, phase III trial of enzastaurin versus placebo in patients achieving remission after first‐line therapy for high‐risk diffuse large B‐cell lymphoma. J Clin Oncol. 2016;34(21):2484‐2492. 27217449 10.1200/JCO.2015.65.7171

[20] Thieblemont C , Tilly H , Gomes da Silva M , et al. Lenalidomide maintenance compared with placebo in responding elderly patients with diffuse large B‐cell lymphoma treated with first‐line rituximab plus cyclophosphamide, doxorubicin, vincristine, and prednisone. J Clin Oncol. 2017;35(22):2473‐2481. 28426350 10.1200/JCO.2017.72.6984

[21] Witzig TE , Tobinai K , Rigacci L , et al. Adjuvant everolimus in high‐risk diffuse large B‐cell lymphoma: final results from the PILLAR‐2 randomized phase III trial. Ann Oncol. 2018;29(3):707‐714. 29253068 10.1093/annonc/mdx764

[22] Schmitz N , Nickelsen M , Ziepert M , et al. Conventional chemotherapy (CHOEP‐14) with rituximab or high‐dose chemotherapy (MegaCHOEP) with rituximab for young, high‐risk patients with aggressive B‐cell lymphoma: an open‐label, randomised, phase 3 trial (DSHNHL 2002‐1). Lancet Oncol. 2012;13(12):1250‐1259. 23168367 10.1016/S1470-2045(12)70481-3

[23] Cortelazzo S , Tarella C , Gianni AM , et al. Randomized trial comparing R‐CHOP versus high‐dose sequential chemotherapy in high‐risk patients with diffuse large B‐cell lymphomas. J Clin Oncol. 2016;34(33):4015‐4022. 28199143 10.1200/JCO.2016.67.2980

[24] Lamy T , Damaj G , Soubeyran P , et al. R‐CHOP 14 with or without radiotherapy in nonbulky limited‐stage diffuse large B‐cell lymphoma. Blood. 2018;131(2):174‐181. 29061568 10.1182/blood-2017-07-793984 PMC5757680

[25] Davies AJ , Barrans S , Stanton L , et al. Differential efficacy from the addition of bortezomib to R‐CHOP in diffuse large B‐cell lymphoma according to the molecular subgroup in the REMoDL‐B study with a 5‐year follow‐up. J Clin Oncol. 2023;41(15):2718‐2723. 36972491 10.1200/JCO.23.00033 PMC10414744

[26] Seymour JF , Pfreundschuh M , Trnĕný M , et al. R‐CHOP with or without bevacizumab in patients with previously untreated diffuse large B‐cell lymphoma: final MAIN study outcomes. Haematologica. 2014;99(8):1343‐1349. 24895339 10.3324/haematol.2013.100818 PMC4116833

[27] Younes A , Sehn LH , Johnson P , et al. Randomized phase III trial of ibrutinib and rituximab plus cyclophosphamide, doxorubicin, vincristine, and prednisone in non‐germinal center B‐cell diffuse large B‐cell lymphoma. J Clin Oncol. 2019;37(15):1285‐1295. 30901302 10.1200/JCO.18.02403 PMC6553835

[28] Lugtenburg P , Avivi I , Berenschot H , et al. Efficacy and safety of subcutaneous and intravenous rituximab plus cyclophosphamide, doxorubicin, vincristine, and prednisone in first‐line diffuse large B‐cell lymphoma: the randomized MabEase study. Haematologica. 2017;102(11):1913‐1922. 28935843 10.3324/haematol.2017.173583 PMC5664395

[29] Sehn LH , Martelli M , Trněný M , et al. A randomized, open‐label, phase III study of obinutuzumab or rituximab plus CHOP in patients with previously untreated diffuse large B‐Cell lymphoma: final analysis of GOYA. J Hematol Oncol. 2020;13(1):71. 32505213 10.1186/s13045-020-00900-7 PMC7276080

[30] Dornan D , Bennett F , Chen Y , et al. Therapeutic potential of an anti‐CD79b antibody‐drug conjugate, anti‐CD79b‐vc‐MMAE, for the treatment of non‐Hodgkin lymphoma. Blood. 2009;114(13):2721‐2729. 19633198 10.1182/blood-2009-02-205500

[31] Polson AG , Yu SF , Elkins K , et al. Antibody‐drug conjugates targeted to CD79 for the treatment of non‐Hodgkin lymphoma. Blood. 2007;110(2):616‐623. 17374736 10.1182/blood-2007-01-066704

[32] Tilly H , Morschhauser F , Sehn LH , et al. Polatuzumab vedotin in previously untreated diffuse large B‐cell lymphoma. N Engl J Med. 2022;386(4):351‐363. 34904799 10.1056/NEJMoa2115304 PMC11702892

[33] Zheng B , Fuji RN , Elkins K , et al. In vivo effects of targeting CD79b with antibodies and antibody‐drug conjugates. Mol Cancer Ther. 2009;8(10):2937‐2946. 19808977 10.1158/1535-7163.MCT-09-0369

[34] Pfeifer M , Zheng B , Erdmann T , et al. Anti‐CD22 and anti‐CD79B antibody drug conjugates are active in different molecular diffuse large B‐cell lymphoma subtypes. Leukemia. 2015;29(7):1578‐1586. 25708834 10.1038/leu.2015.48

[35] National Comprehensive Cancer Network . National Comprehensive Cancer Network (NCCN) Guidelines ‐ B‐Cell Lymphomas 2023; Version 1.

[36] Maurer MJ , Ghesquières H , Jais JP , et al. Event‐free survival at 24 months is a robust end point for disease‐related outcome in diffuse large B‐cell lymphoma treated with immunochemotherapy. J Clin Oncol. 2014;32(10):1066‐1073. 24550425 10.1200/JCO.2013.51.5866 PMC3965261

[37] Shi Q , Schmitz N , Ou FS , et al. Progression‐free survival as a surrogate end point for overall survival in first‐line diffuse large B‐cell lymphoma: an individual patient‐level analysis of multiple randomized trials (SEAL). J Clin Oncol. 2018;36(25):2593‐2602. 29975624 10.1200/JCO.2018.77.9124 PMC6532366

[38] Maurer MJ , Habermann TM , Shi Q , et al. Progression‐free survival at 24 months (PFS24) and subsequent outcome for patients with diffuse large B‐cell lymphoma (DLBCL) enrolled on randomized clinical trials. Ann Oncol. 2018;29(8):1822‐1827. 29897404 10.1093/annonc/mdy203 PMC6096732

[39] Jakobsen LH , Bøgsted M , Brown PN , et al. Minimal loss of lifetime for patients with diffuse large B‐cell lymphoma in remission and event free 24 months after treatment: a Danish population‐based study. J Clin Oncol. 2017;35(7):778‐784. 28095160 10.1200/JCO.2016.70.0765

[40] Lee L , Wang L , Crump M . Identification of potential surrogate end points in randomized clinical trials of aggressive and indolent non‐Hodgkin's lymphoma: correlation of complete response, time‐to‐event and overall survival end points. Ann Oncol. 2011;22(6):1392‐1403. 21266519 10.1093/annonc/mdq615 PMC3101365

[41] Zhu J , Yang Y , Tao J , et al. Association of progression‐free or event‐free survival with overall survival in diffuse large B‐cell lymphoma after immunochemotherapy: a systematic review. Leukemia. 2020;34(10):2576‐2591. 32651542 10.1038/s41375-020-0963-1 PMC7515849

[42] Nowakowski GS , Chiappella A , Gascoyne RD , et al. ROBUST: a phase III study of lenalidomide plus R‐CHOP versus placebo plus R‐CHOP in previously untreated patients with ABC‐type diffuse large B‐cell lymphoma. J Clin Oncol. 2021;39(12):1317‐1328. 33621109 10.1200/JCO.20.01366 PMC8078325

[43] Tierney JF , Stewart LA , Ghersi D , Burdett S , Sydes MR . Practical methods for incorporating summary time‐to‐event data into meta‐analysis. Trials. 2007;8:16. 17555582 10.1186/1745-6215-8-16 PMC1920534

[44] Fu Z , Li S , Han S , Shi C , Zhang Y . Antibody drug conjugate: the "biological missile" for targeted cancer therapy. Signal Transduct Target Ther. 2022;7(1):93. 35318309 10.1038/s41392-022-00947-7 PMC8941077

[45] Varma G , Goldstein J , Advani RH . Novel agents in relapsed/refractory diffuse large B‐cell lymphoma. Hematol Oncol. 2023;41(Suppl 1):92‐106. 37294966 10.1002/hon.3143

[46] Caimi PF , Ai W , Alderuccio JP , et al. Loncastuximab tesirine in relapsed or refractory diffuse large B‐cell lymphoma (LOTIS‐2): a multicentre, open‐label, single‐arm, phase 2 trial. Lancet Oncol. 2021;22(6):790‐800. 33989558 10.1016/S1470-2045(21)00139-X

[47] Abramson JS , Palomba ML , Gordon LI , et al. Lisocabtagene maraleucel for patients with relapsed or refractory large B‐cell lymphomas (TRANSCEND NHL 001): a multicentre seamless design study. Lancet. 2020;396(10254):839‐852. 32888407 10.1016/S0140-6736(20)31366-0

[48] Locke FL , Miklos DB , Jacobson CA , et al. Axicabtagene ciloleucel as second‐line therapy for large B‐cell lymphoma. N Engl J Med. 2022;386(7):640‐654. 34891224 10.1056/NEJMoa2116133

[49] Viardot A , Goebeler ME , Hess G , et al. Phase 2 study of the bispecific T‐cell engager (BiTE) antibody blinatumomab in relapsed/refractory diffuse large B‐cell lymphoma. Blood. 2016;127(11):1410‐1416. 26755709 10.1182/blood-2015-06-651380 PMC4797019

[50] Hutchings M , Morschhauser F , Iacoboni G , et al. Glofitamab, a novel, bivalent CD20‐targeting T‐cell‐engaging bispecific antibody, induces durable complete remissions in relapsed or refractory B‐cell lymphoma: a phase I trial. J Clin Oncol. 2021;39(18):1959‐1970. 33739857 10.1200/JCO.20.03175 PMC8210975

[51] Lin JK , Muffly LS , Spinner MA , Barnes JI , Owens DK , Goldhaber‐Fiebert JD . Cost effectiveness of chimeric antigen receptor T‐cell therapy in multiply relapsed or refractory adult large B‐cell lymphoma. J Clin Oncol. 2019;37(24):2105‐2119. 31157579 10.1200/JCO.18.02079

