# Supplementary material for: Prediction of 5‐year overall survival of diffuse large B‐cell lymphoma on the pola‐R‐CHP regimen based on 2‐year event‐free survival and progression‐free survival
Source: Cancer Med. 2024 Jan 5;13(1):e6899. doi: 10.1002/cam4.6899 (PMC10807604; doi:10.1002/cam4.6899)
Supplement: Supplementary file 1 — Figure S1. [file CAM4-13-e6899-s002.pdf]

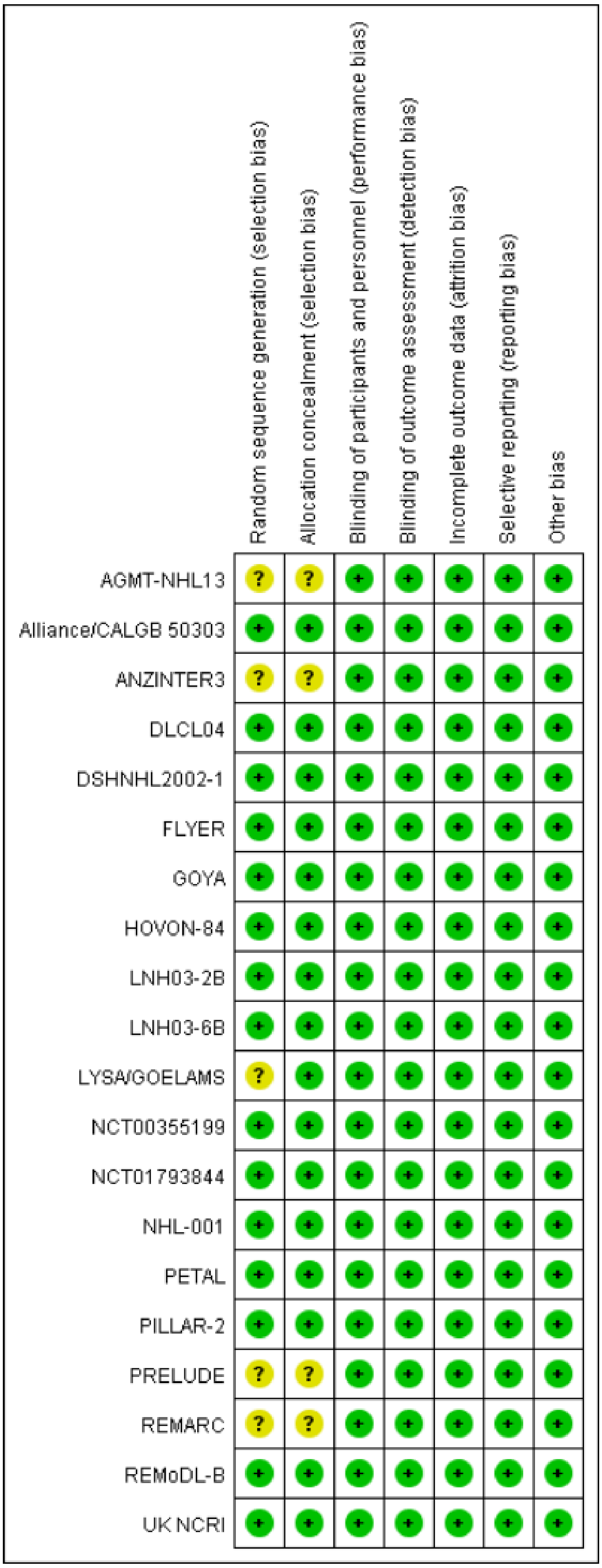

Supplemental Fig. 1 Summary of risk of bias in RCTs.
