# Supplementary material for: Prediction of 5‐year overall survival of diffuse large B‐cell lymphoma on the pola‐R‐CHP regimen based on 2‐year event‐free survival and progression‐free survival
Source: Cancer Med. 2024 Jan 5;13(1):e6899. doi: 10.1002/cam4.6899 (PMC10807604; doi:10.1002/cam4.6899)
Supplement: Supplementary file 2 — Table S1. [file CAM4-13-e6899-s001.docx]

**Supplemental Table 1.** Reasons for exclusion of phase III randomized controlled trials.

| **Trial** | **Inclusion Criteria** | **Median FU (y)** | **Treatment** | **Reasons for exclusion** |
| --- | --- | --- | --- | --- |
| **R-CHOP (like) vs CHOP (like) (n = 4)** | | | | |
| LNH-98.5 (Coiffier, 2010)^1^ | Age 60-80; stage II-IV; PS ≤2 | 10 | R-CHOP | CHOP regimen as standard treatment arm. |
|  |  |  | **CHOP** |  |
| MInT (Pfreundschuh, 2011) ^2^ | Age 18-60; aaIPI ≤1; stage II-IV or bulky stage I | 6 | R-CHOP-like | CHOP regimen as standard treatment arm. |
|  |  |  | **CHOP-like** |  |
| ECOG4494/CALGB9793 (Habermann, 2006) ^3^ | 1^st^ randomization: age ≥60; all stage; PS ≤3 | 3.5 | R-CHOP | CHOP regimen as standard treatment arm. |
|  |  |  | **CHOP** |  |
| RICOVER-60 (Pfreundschuh, 2008) ^4^ | Age 61-80; all stages | 2.9 | 8R-CHOP | CHOP regimen as standard treatment arm. |
|  |  |  | 6R-CHOP |  |
|  |  |  | 8CHOP |  |
|  |  |  | **6CHOP** |  |
| **R-CHOP (like) vs R + intensified/de-escalated chemotherapy (n = 1)** | | | | |
| ROBUST (Nowakowski, 2021)^42^ | Age 18-80; ABC-type DLBCL; PS ≤2; stage II-IV; IPI ≥2 | 2.3 | R-CHOP+Lenalidomide | Due to the short follow-up time, only a few number of patients at risk in 5 years. Data not available for 5-year OS rates. |
|  |  |  | **R-CHOP** |  |
| **R-CHOP (like) chemotherapy followed by maintenance/consolidation therapy (n = 1)** | | | | |
| ECOG4494/CALGB9793 (Habermann, 2006)^3^ | 2^nd^ randomization: age ≥60; all stage; PS ≤3; CR/PR | 3.5 | R maintenance | Due to the short follow-up time, only a few number of patients at risk in 5 years. Data not available for 5-year OS rates. |
|  |  |  | **Observation** |  |
| **R-CHOP + novel targeted drug (n =2)** | | | | |
| MAIN (Seymour, 2014)^26^ | Age ≥18; all stages | 2 | RA-CHOP | Due to the short follow-up time, only a few number of patients at risk in 5 years. Data not available for 5-year OS rates. |
|  |  |  | **R-CHOP** |  |
| PHOENIX (Younes, 2019) ^27^ | Age ≥18; non-GCB; stage II-IV; R-IPI ≥1; PS ≤2 | 2.9 | R-CHOP+ibrutinib | Due to the short follow-up time, only a few patients at risk in 5 years. Data not available for 5-year OS rates. |
|  |  |  | **R-CHOP** |  |
| **Anti-CD20 monoclonal antibody study (n = 1)** | | | | |
| MabEase (Lugtenburg, 2017)^28^ | Age 18-80; ≥ 1.5 cm; IPI 0 (≥7.5 cm) or 1-5; PS ≤2 | 2.9 | R(SC)+CHOP | Due to the short follow-up time, only a few number of patients at risk in 5 years. Data not available for 5-year OS rates. |
|  |  |  | **R(IV)+CHOP** |  |

The standard arm is labeled in bold.

**Abbreviations:** **aaIPI,** age-adjusted International Prognostic Index; **CR,** complete response; **DLBCL,** diffuse large B-cell lymphoma; **IPI,** International Prognostic Index; **PR,** partial response; **PS,** performance status; **FU,** median follow-up; **y,** year.

**Trials:** **LNH98-5,** Lymphome Non Hodgkinien study 98-5; **MInT,** MabThera International Trial; **ECOG4494/CALGB9793,** The Eastern Cooperative Oncology Group 4494/Cancer and Leukemia Group B 9793; **RICOVER-60,** rituximab with cyclophosphamide, doxorubicin, vincristine, and prednisone age >60 years.

**Chemotherapy regimens:** **R-CHOP,** rituximab, cyclophosphamide, doxorubicin, vincristine, and prednisone.
